# The Story of the Hospitals

**Published:** 1886-10-23

**Authors:** 


*(To be continued.)*

### The Story of the Hospitals.

(*Continued from page* 11.)

**Onward!** The London Hospital — the great hospital for the teeming population of East London—presents a history which is paralleled by few institutions. From the day of its foundation, its career has been one of ceaseless and rapid progress. The watchword of its Governors has been "Onward!" It has surmounted many a periodic wave of depression. Over and over again an impoverished exchequer has called forth renewed exertions, and each appeal to Christian philanthropy has been met by the public with a liberal response. Despite the additions to the hospital which I mentioned in my former article, the increasing population of Whitechapel and the neighbourhood soon made the supply inadequate to the want. The Governors saw with deep regret some thousand urgent cases annually turned away. In 1831 a further extension of the hospital was determined upon, and accommodation was accordingly made for one hundred more beds. In 1840 a centenary festival was held, and the occasion was deemed an appropriate one for a further appeal. This was made, and resulted in a collection of £12,000. The East Wing, with seventy-four beds, was now built. Another twenty years sped by, and once more the constant growth of the locality rendered still further additions imperative. Once again the generous public were appealed to, and the appeal was met by a contribution of £34,000.

**A Memorable Year.** On 4th July 1864, the Princess of Wales laid the foundation stone of the new "Alexandra Wing," and, as the building was approaching completion, preparations were in active progress for the interesting inaugural ceremony. "Man proposes, but God disposes." So it was at the opening of the "Alexandra Wing." It had been arranged that this should take place in July 1866, but just at this period a terrible scourge swept over the East-end. At the moment when all was to have been rejoicing at the happy completion of the work to which the Princess of Wales had two years before graciously set her hand, the new building was crowded, at a few hours' notice, with the unhappy victims of cholera. The year 1866 was a memorable one in the annals of the London Hospital. The magnificent service which this institution rendered to suffering humanity in this terrible hour of affliction was acknowledged by the public throughout the length and breadth of the land in a spirit of noble generosity and national gratitude. The donations this year amounted to £28,000, thereby relieving the Governors of all fears of financial embarrassment. During the epidemic, eight hundred and sixty-five patients were admitted into the Cholera Wards, while nearly thirteen thousand out-patients were treated. The total number of in-patients in this year was thus increased to five thousand one hundred and nine, while the out-patients numbered forty-five thousand two hundred and six.

**A Continuous Development.** The subsequent history of the London Hospital is one of continuous development and improvement. Special wards—such as the ophthalmic, children's medical, and isolation—have been opened. The Medical Waiting Hall has been much enlarged, and new rooms have been added for the medical and surgical examination of out-patients. In 1872 a further addition of two hundred beds was made to the institution, and in the following year a special fund was established to meet the increasing expenses of extension and maintenance. The Grocers' Company having made in this emergency a munificent gift of £20,000, the new building, opened by Her Majesty in 1876, was styled "The Grocers' Company Wing." This last addition has raised the total complement of beds to seven hundred and ninety, thus making the London Hospital by far the largest in the United Kingdom.

**Its Work.** Having now briefly sketched the history of the London Hospital from its foundation, 146 years ago, to the present time, I will turn for a few moments to the work which this vast establishment is daily carrying on. The London Hospital is now virtually a free establishment, for the check which has been wisely placed upon the promiscuous admission of out-patients in no way destroys, or even diminishes, the principle of which I speak. Twenty years ago things were different. It was then practically a proprietary establishment, the great majority of the patients being admitted on the recommendation of the Governors. Many cases of a minor character were admitted then which would not be received now, while, on the other hand, the delay in the admission of a patient sometimes resulted in the aggravation of his malady. To-day, however, a far better system prevails at the London, which renders it an institution devoted to the service of the public, and especially to the service of the poor. The Governors, it is true, still enjoy certain privileges of introduction, but the fact that nearly three-fourths of the in-patients are admitted on the advice of the medical and surgical officers under the "Extra Case" system, shows the free and public character of the institution. The most marvellous growth of the hospital has been in the past forty years, during which period the in-patient work has more than doubled itself. While in 1844 the admissions numbered 3,691, they reached in 1885 no less than 8,106. Despite the careful reorganisation of the out-patient department, the cases treated last year reached nearly 68,000.

**Wanted £20,000.** To meet an essential expenditure of about £49,000 per annum, the hospital has a fixed income only to the extent of £15,562, derived from funded property, house rent, etc. When we remember the fact that the hospital is situated in the midst of a population of the very poorest class, it is obvious that the big deficit which has every year to be met must come from "outside." But the House Governor of the London Hospital has faith in the perennial flow of British charity, for, after telling me about the money troubles of the hospital, he added, "I believe while we, on our part, do what we can to keep up our resources, and conduct the institution on the lines of strict economy, so long shall we be properly supported."

(*To be continued.*)

---

A Strange Patient.—Dr. James Hamilton was once summoned by a lady of rank to attend her pet *monkey*, the little favourite having almost suffocated itself with its morning feed. When the doctor entered the room he saw only her ladyship, her young son (a lad of ten, who was most absurdly dressed), and his "patient." Looking at each of the two latter, he calmly inquired of her ladyship, "Which is the monkey?"

